# Characterization and Dynamics of the Gut Microbiota in Rice Fishes at Different Developmental Stages in Rice-Fish Coculture Systems

**DOI:** 10.3390/microorganisms10122373

**Published:** 2022-11-30

**Authors:** Ling Tao, Jie Chai, Hongyi Liu, Wenhao Huang, Yan Zou, Mengling Wu, Buqing Peng, Qiong Wang, Keyi Tang

**Affiliations:** 1 College of Life Sciences, Sichuan Normal University, Chengdu 610066, China; 2 Chongqing Academy of Animal Sciences, Chongqing 402460, China; 3 College of Biology and the Environment, Nanjing Forestry University, Nanjing 210037, China

**Keywords:** rice-fish system, 16S rRNA gene, microbial ecology, carp, growth stages

## Abstract

The rice-fish system (RFS), a traditional coculture farming model, was selected as a “globally important agricultural heritage system.” Host-associated microbiota play important roles in development, metabolism, physiology, and immune function. However, studies on the gut microbiota of aquatic animals in the RFS are scarce, especially the lack of baseline knowledge of the dynamics of gut microbial communities in rice fish during different developmental stages. In this study, we characterized the microbial composition, community structure, and functions of several sympatric aquatic animals (common carp (*Cyprinus carpio*), crucian carp (*Carassius carassius*), and black-spotted frogs (*Pelophylax nigromaculatus*)), and the environment (water) in the RFS using 16S rRNA gene sequencing. Moreover, we investigated stage-specific signatures in the gut microbiota of common carp throughout the three developmental stages (juvenile, sub-adult, and adult). Our results indicated that the Fusobacteriota, Proteobacteria, and Firmicutes were dominant gut microbial phyla in rice fish. The differences in gut microbial compositions and community structure between the three aquatic species were observed. Although no significant differences in alpha diversity were observed across the three developmental stages, the microbial composition and community structure varied with development in common carp in the RFS, with an increase in the relative abundance of Firmicutes in sub-adults and a shift in the functional features of the community. This study sheds light on the gut microbiota of aquatic animals in the RFS. It deepens our understanding of the dynamics of gut microflora during common carp development, which may help improve aquaculture strategies in the RFS.

## 1. Introduction

The rice-fish system (RFS), a “globally important agricultural heritage system” [1], is an integrated agri-aquaculture system (IAAS) that combines rice cultivation with aquaculture [2,3]. Compared to rice monoculture, a wide range of aquatic species (i.e., fish, carp, crabs, eel, and softshell turtles) are used in the RFS [4,5]. Rice and various animal species raised in the same system influence each other to balance the role of the rice-field ecosystem [6,7]. The RFS can harvest both highly stable aquatic products and rice [8], thereby increasing farmers’ income [9]. In addition, this system can effectively reduce the use of pesticides and chemical fertilizers because rice fishes can prey on pests and aquatic plants (e.g., duckweed) and their excreta can enhance soil fertility [1,10,11].

Several commercial fishes (e.g., common carp (*Cyprinus carpio*) and crucian carp *(Carassius carassius*)) are commonly used species raised in RFS [12]. Paddy fields contain various abundant aquatic plants and animals, and rice fishes can forage the natural diets (such as insects, weeds, macro-algae, and phytoplankton), resulting in a great reduction in pests and weeds [13,14]. Meanwhile, rice fishes are able to convert foods into biomass and waste, returning it to the environment and achieving the purpose of increasing rice yield [5]. Most studies on rice-fish systems have focused on the ecology of aquatic animals [15,16,17], biodiversity conservation [1,5,18], and economic value [3,9]. In addition, an increasing number of studies on the gut microbial community of aquaculture animals raised in the RFS have been conducted using both DGGE technology and 16S rRNA sequencing, such as in the rice field eel (*Monopterus albus*) [4,19], carp [13,20], loach [21], and yellow catfish [22].

Microbes colonize the gastrointestinal (GI) tract of vertebrates and play an important role in maintaining host growth and development [23,24], stimulating intestinal cell proliferation [25], nutrient absorption [26,27], disease prevention [28,29], immunity, and health [30,31]. A healthy gut microbial community is both resilient and flexible [32]. In the fish gut ecosystem, bacteria are assumed to directly colonize the GI tract of fish from the surrounding environmental water [33]. In addition to the surrounding environment, host-related factors (e.g., diet [34,35,36], host genetics [37,38,39], and physiological characteristics [37,40]) may also shape fish-associated microbial communities. In addition, during host development, gut microbiota can profoundly influence the host by harvesting energy from the diet, conferring resistance to pathogens, and stimulating the development of gut function and the immune system [34,41,42]. Therefore, maintaining the dynamic stability of the microbial community is crucial for maintaining the intestinal health of fish. Previous studies have demonstrated that the ecological and physiological development of the host has a significant impact on the microbial community in zebrafish (*Danio rerio*) [43,44,45], cod (*Gadus morhua*) [46], gibel carp (*Carassius auratus*) [33], Southern catfish (*Silurusasotus*) [47], Atlantic salmon (*Salmo salar*) [48], channel catfish (*Ictalurus punctatus*) [49], and seabream (*Pagrus major*) [50]. As a result, it is important to characterize how and which microbes vary over the host’s ontogeny in the RFS, as they can have large effects on the development and growth of rice fish. However, studies of the dynamics of gut microbiota in rice fish throughout the different developmental stages remain poorly understood. In RFS, the black-spotted frog (*Pelophylax nigromaculatus*) is often sympatric with rice fish (i.e., common carp and crucian carp), which experience similar environmental conditions and nutritionally similar diets (prey on rice pests) [51]. Previous studies have indicated that different species reared in the same water environment vary in their gut microbiota [52,53], suggesting that gut microbial communities exhibit strong host specificity. Recent studies in rice field fish have generated important data regarding the composition of the gut microbiota [12,20]; however, little is known about the similarity or heterogeneity of the intestinal microflora of sympatric aquatic animals in RFS. To improve existing coculture strategies in RFS, it is also essential to investigate the gut microbial ecology of rice fish.

In this study, two commercial fishes (common carp and crucian carp) were partnered with rice plants during the entire rice-growing period (130–150 days) and harvested in September in Sichuan Province, China. We collected colonic contents from common carp at three different developmental stages (juvenile (May), sub-adult (July), and adult (September)). In addition, we also collected water samples and colonic contents from sympatric crucian carp and black-spotted frogs at the same time as we obtained the sub-adult samples. 16S rRNA gene amplicon high-throughput sequencing was used to (1) characterize the intestinal bacterial community composition and diversity of these aquatic animals and (2) explore temporal changes in the membership and structure of gut microbiota in common carp at three different developmental stages. The present study may enhance our understanding of the gut microbiota of common aquatic animals in RFS and provide a theoretical basis for healthy coculture of rice fish in rice paddy fields.

## 2. Materials and Methods

### 2.1. Co-Culturing and Sample Collection

The experimental paddy field (30°23′54″ N, 103°57′55″ E) was located in Shuangliu County, Chengdu, Sichuan Province (China), the size of which was approximately 660 m^2^. Common carp and crucian carp are traditionally raised together in RFS. The fries of common carp and crucian carp were simultaneously released into the rice field one week after rice was transplanted in late May 2021. The growing season for rice carp is usually from late May to September (approximately 110 days). Carps do not feed on rice plants. No chemicals were used to control weeds, pests, or diseases during the entire coculture process. Paddy fields contain a wide range of plants and animals, allowing rice fishes to have both a plant and animal diet. These aquaculture animals can feed on a natural diet with the aim of providing healthy aquatic products for RFS. Nineteen common carp samples were collected in late May (juveniles, *n* = 8), July (subadults, *n* = 6), and September (adults, *n* = 5). Black-spotted frogs and two species of rice fish are sympatric in rice paddies with similar environmental conditions. To compare the gut microbial communities of sympatric frogs and fishes, specimens of black-spotted frogs (*n* = 6) and crucian carp (*n* = 3) were also collected in July of the same year. Water samples were obtained from different depths in paddy fields at the same time (July) and stored in sterile EP tubes. The collected fish, black-spotted frogs, and water samples were transported to the laboratory at the College of Life Sciences, Sichuan Normal University.

After anesthesia, all specimens were dissected on a clean bench, and the intestinal tracts were removed from the abdominal cavity. The colon contents of each individual were squeezed out and collected separately using fiber swabs to collect clinical microorganisms (CY-98000). All samples were stored at −80 °C for DNA extraction. This study was performed in accordance with recommendations for animal care and ethics in China. All samples were collected following the guidelines and approval of the Animal Ethics and Welfare Committee of Sichuan Normal University (2020LS012).

### 2.2. DNA Extraction and 16S rRNA Gene Sequencing

Total bacterial DNA was extracted using the QIAamp Fast DNA Stool Mini Kit (Qiagen, Valencia, CA, USA) and stored at −80 °C for analysis of the microbiota. The 16S rRNA gene was amplified using the 341f/806r primer set (341f, 5′-CCTAYGGGRBGCASCAG-3′, and 806r, 5′-GGACTACNNGGGTATCTAAT-3′), which targets the V3–V4 hypervariable region of the gene. The parameters and experimental methods of PCR reactions and amplification have been described in our previous study [54]. The PCR mix contained 15 μL of Phusion Master Mix, 1 μL of each primer, 3 μL of ultrapure water, and 10 μL of DNA for a final volume of 30 μL. The PCR cycle conditions were as follows: preheating at 98 °C for 1 min; 30 cycles of 98 °C for10 s, 50 °C for 30 s, and 72 °C for 30 s; final extension at 72 °C for 5 min. All PCRs were performed using Phusion^®^ High-Fidelity PCR Master Mix (New England Biolabs, Ipswich, MA, USA) according to the manufacturer’s instructions. The products were detected by electrophoresis on 2% (*w*/*v*) agarose gel for verification. Sequencing libraries were generated using the NEBNext^®^ Ultra^TM^II DNA Library Prep Kit, as recommended by the manufacturer. The constructed libraries were quantified by Qubit and Q-PCR. PCR products were sequenced on a NovaSeq 6000 sequencing platform (2 × 250 bp paired-end reads) by the Novogene Bioinformatics Technology Corporation (Beijing, China).

### 2.3. Bioinformatics and Amplicon Sequencing Data Analyses

The reads of the samples were assembled using FLASH (V1.2.11, http://ccb.jhu.edu/software/FLASH/, accessed on 9 August 2021) [55] to obtain raw tags. Then, the raw tags were quality-controlled using fastp software to obtain high-quality clean tags. Clean tags were aligned to the database using Usearch software to detect and remove chimeras [56]. Noise reduction was performed using the DADA2 module in the QIIME2 [57] software, and sequences with an abundance of less than five were filtered out, thus obtaining the final amplicon sequence variants (ASVs). Subsequently, the resulting ASVs were aligned to the SILVA database (version 138.1) using the classify-sklearn module in QIIME2 software [58,59] to obtain taxonomic information for each ASV. To evaluate the species diversity and richness of the microbial communities of the three aquatic animals and water samples, alpha diversity indices (including observed OTUs, Chao1, Shannon, and Simpson) were calculated using the QIIME2. Significant differences between alpha diversity indices were evaluated using the Wilcoxon test with R software v.4.1.1. [60]. A rarefaction curve was used to describe the variation in sequencing depth and diversity for each sample. We then used beta diversity to analyze the microbial community structure among the different species. Beta diversity was analyzed based on weighted UniFrac and unweighted UniFrac distances using QIIME2 and visualized using two-dimensional principal coordinate analysis (PCoA). Analysis of similarity (ANOSIM) was performed to test for differences in bacterial communities between different groups based on weighted UniFrac and unweighted Unifrac distance matrices using the R “Vegan” package [61]. We also compared the relative abundance of bacterial taxa based on the linear discriminant analysis (LDA) effect size (LEfSe) method using the LEfSe software [62]. Statistically significant differences in the relative abundance of bacterial taxa between groups were assessed using the MetaStats test [63]. Boxplots, bar charts, PCoA, and heatmaps were generated using the “ggplot2” package of R software v.4.1.1. and pie charts were created by Tableau (2019). 2 software. Tax4Fun [64] was used to predict the functions of each ASV based on the Silva database. Molecular functions were predicted and summarized in the Kyoto Encyclopedia of Genes and Genomes (KEGG) pathway functions and different levels of pathway categories [65].

## 3. Results

### 3.1. Overview of Sequencing Data

A total of 3,903,082 high-quality clean reads, with an average length of 413 bp, was obtained for all samples. A total of 6973 ASVs were obtained, and the number of ASVs detected per sample ranged from 215 to 2096 (more details on sequencing are shown in (Table S1). A total of 113 core ASVs were shared in three different developmental stages (Figure S1A) in the common carp, with 418 shared ASVs in the common carp and crucian carp (Figure S1B). The rarefaction curve of bacterial ASV detected from the sample was relatively high and flat as the number of sequences increased (Figure S1C), indicating that the sequencing depth was sufficient to capture the diversity of the microbial community. The coverage index ranged from 0.99 to 1 (Table S2), indicating that the sequences were sufficient to fully demonstrate the species richness of the samples.

### 3.2. Gut Microbial Community of Three Sympatric Aquatic Animals

#### 3.2.1. Characteristics of Microbial Composition in Three Sympatric Species

**Figure 1:**
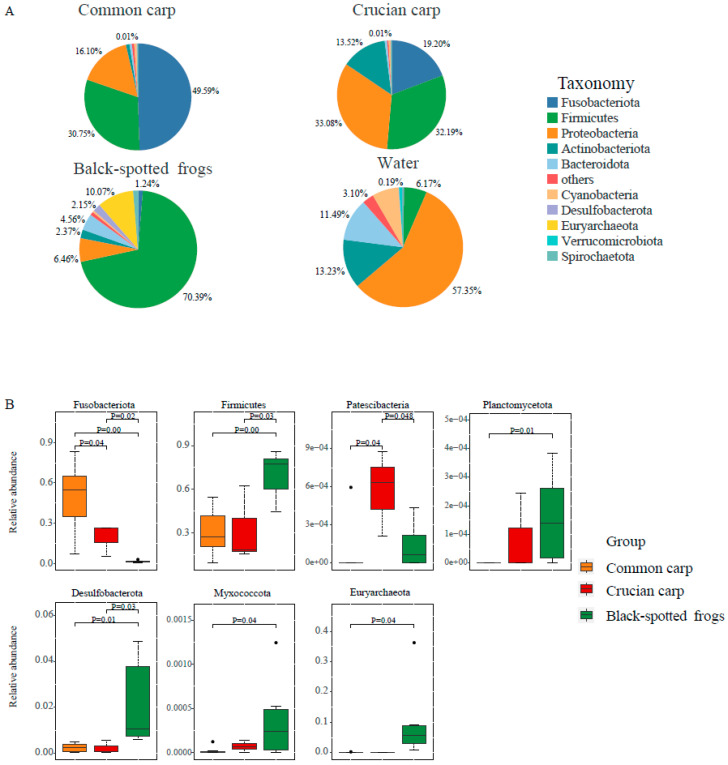
Microbial composition of the common carp, crucian carp, black-spotted frogs, and water samples. (**A**) The relative abundance of the top 10 bacterial phyla in the GI tracts of common carp, crucian carp, black-spotted frog, and water environment. (**B**) Box-and-whisker plots for the relative abundance of six bacterial phyla with significant differences (*p* < 0.05) between the four groups based on the Metastat test.

The microbial compositions at the phylum level in the three sympatric aquatic species and water samples collected in July are shown in the pie plot (Figure 1A). Fusobacteriota, Proteobacteria, Firmicutes, and Actinobacteria were the four most dominant phyla within the gut microbial community of the two rice fishes, accounting for more than 98% of all reads (Figure 1A and Table S3). The gut microbiota of the common carp comprised 26 phyla, 59 classes, 146 orders, 244 families, and 507 genera (Table S1). The most abundant bacterial phylum in the common carp was Fusobacteriota (49.59 ± 11.24%), followed by Firmicutes (30.75 ± 6.89%) and Proteobacteria (16.10 ± 6.05%) (Figure 1A and Table S3). Gut microbes in the GI tracts of crucian carp were assigned to 22 phyla, 39 classes, 103 orders, 171 families, and 292 genera (Table S1). The crucian carp harbored a prominent community of Proteobacteria (33.08 ± 13.32%), Firmicutes (32.19 ± 15.08%), Fusobacteriota (19.20 ± 7.01%), and Actinobacteria (13.52 ± 11.13%). The overall gut microbiota of black-spotted frogs was characterized by a high prevalence of Firmicutes, Euryarchaeota, Proteobacteria, and Bacteroidota, which together made up the vast majority (>90%) (Figure 1A and Table S3). The Firmicutes (70.39 ± 16.77%) was the highest-represented phylum, followed by Euryarchaeota (10.07 ± 5.37%), which was not detected in the water environment, with combined relative abundances accounting for up to 80% of the overall microflora in black-spotted frogs (Figure 1A and Table S3). The microbial community in water samples was dominated by Proteobacteria (57.35 ± 1.12%), Bacteroidota (11.49 ± 0.65%), Actinobacteriota (13.23 ± 1.51%), Cyanobacteria (7.17 ± 2.68%), and Firmicutes (6.17 ± 3.38%). Significant differences in the composition of the dominant phyla (i.e., Proteobacteria, Actinobacteriota, Cyanobacteria, and Verrucomicrobiota) were detected between the three aquatic animals and water samples (Figure 1A and Table S3). Proteobacteria, Actinobacteriota, Cyanobacteria, and Bacteroidota phyla showed significantly higher relative abundances in water samples than in common carp (Figure 1A and Table S3).

We also observed interspecies differences in the microbial composition at the phylum level using the MetaStats test (Figure 1B and Table S3). For example, the phylum Fusobacteriota, which was dominated by the genus *Cetobacterium*, was detected at a lower relative abundance in black-spotted frogs than in common carp and crucian carp (MetaStats test; *p* = 0.007 and *p* = 0.02, respectively). The phylum Patescibacteria was detected at a higher relative abundance in crucian carp than in black-spotted frogs and common carp (MetaStats test; *p* = 0.048 and *p* = 0.04, respectively) (Figure 1B). Firmicutes and Desulfobacterota were more abundant in black-spotted frogs than in sympatric common and crucian carp (Figure 1B).

At the genus level, the bacterial composition varied among the three sympatric aquatic animals (Table S4). *Cetobacterium* (49.24 ± 11.20%) was detected at the highest relative abundance, followed by *Romboutsia* (15.00 ± 5.50%) and *Aeromonas* (11.51 ± 5.52%) in common carp (Table S4). The most prevalent genus in crucian carp was also *Cetobacterium* (17.95 ± 6.91%), followed by *Romboutsia* (15.70 ± 12.50%), *Aeromonas* (3.00 ± 1.44%), *Vibrio* (10.11 ± 9.75%), *Pseudomonas* (11.80 ± 7.10%), and *Aurantimicrobium* (6.50 ± 5.98%) (Table 1). In the GI tracts of black-spotted frogs, the genera *TC1* (22.68 ± 5.47%), *Mycoplasma* (4.04 ± 3.16%), *Parabacteroides* (2.78 ± 2.25%) were detected at high relative abundances (Table S4). Overall, the genera *Cetobacterium*, *Romboutsia*, and *Aeromonas* were detected in higher relative abundance in common carp and crucian carp than in black-spotted frogs (Table S4). In contrast, the genera *TC1* and *Vibrio* showed a higher relative abundance in black-spotted frogs (Table S4).

Additionally, the LEfSe method was used to identify ASVs differentially represented among the three sympatric species, and 41 taxa with discrepancies in relative abundances were identified with an LDA score > 4 (Figure S2). In total, 9, 10, and 22 biomarkers were identified in the black-spotted frog, crucian carp, and common carp, respectively. The *Mycoplasma* and *Erysipelatoclostridium* genera differed significantly among the three species and were significantly enriched in the common carp. Bacterial taxa from the Methanobacteriaceae family were the top biomarkers distinguishing crucian carp from the other two groups. Desulfitobacteriales was the top family-level biomarker distinguishing black-spotted frogs from all other host groups.

#### 3.2.2. Alpha and Beta Diversity of Gut Microbiota among Three Sympatric Aquatic Species

**Figure 2:**
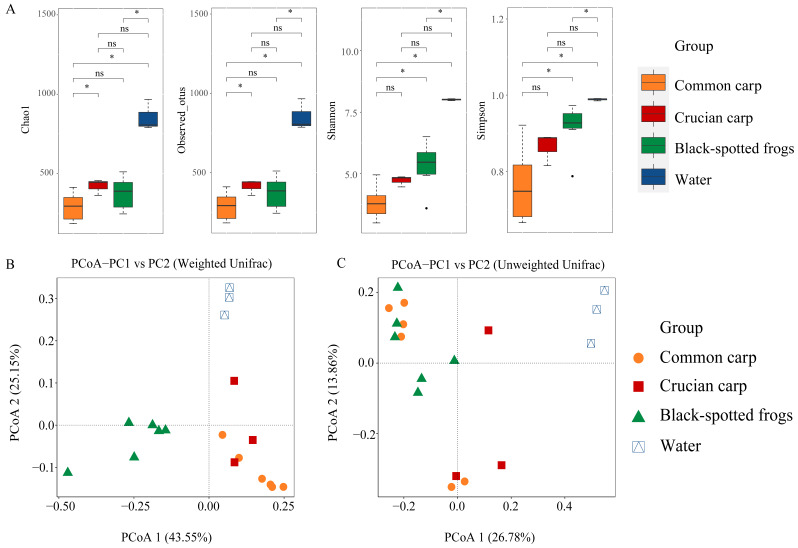
The alpha and beta diversity of microbial communities in common carp, crucian carp, black-spotted frogs, and water samples. (**A**) Box-and-whisker plots for comparison of alpha diversity of microbial communities between common carp, crucian carp, black-spotted frog, and water environment. “*” indicates there are significant differences (*p* < 0.05) between the groups and “ns” means no significant differences. (**B**) Two-dimensional principal coordinate analysis (PCoA) of bacterial communities between the four groups based on weighted UniFrac (**B**) and unweighted UniFrac (**C**) distances. Each symbol corresponds to one sample. The first two principal coordinate (PC) axes are shown.

The Chao1, observed OTUs, Shannon, and Simpson indices were compared between these four groups based on the Wilcoxon rank-sum test (Figure 2A and Table S5). The microbial richness (Chao1 and observed OTUs) and diversity (Shannon and Simpson) in water samples were significantly greater than those in common carp and black-spotted frogs (*p* = 0.02 and 0.02, respectively) (Figure 2A; Table S5), indicating a more diverse microbial community in the water environment. The Shannon and Simpson indices in black-spotted frogs were significantly higher than common carp (*p* = 0.02 and 0.02, respectively). Chao1 and observed OTUs indices of crucian carp were higher than those of common carp (*p* = 0.04 and 0.04, respectively) (Figure 2A; Table S5). However, there was no significant difference in richness and diversity between crucian carp and water and between crucian carp and black-spotted frogs (Figure 2A; Table S5). Gut content samples collected from the common carp exhibited the lowest bacterial richness and diversity (Figure 2A; Table S5).

Based on weighted UniFrac distances, the bacterial communities of rice fish, frogs, and water samples tended to cluster and were separated from each other with some overlap within two fish (Figure 2B), revealing that each group hosted a unique gut microbial community. Based on the unweighted UniFrac distances, the microbiota of common carp and crucian carp did not tightly cluster based on species or water samples. The gut microbiota of black-spotted frogs was clustered relatively closely, with some overlap with common carp (Figure 2C). The clustering pattern was also confirmed by ANOSIM, and the gut microbiota of black-spotted frogs showed significant separation from the bacterial communities of two sympatric rice fishes (ANOSIM, R = 0.372–0.737, *p* = 0.005 and 0.015; Table 1). However, there was no significant difference in the microbial community structure (ANOSIM, R = 0.284, *p* = 0.159; Table 1) between common carp and crucian carp under the weighted UniFrac distances.

### 3.3. Temporal Variations in Gut Microbiota of Common Carp during Different Development Stages

#### 3.3.1. Temporal Changes in Microbial Composition

**Figure 3:**
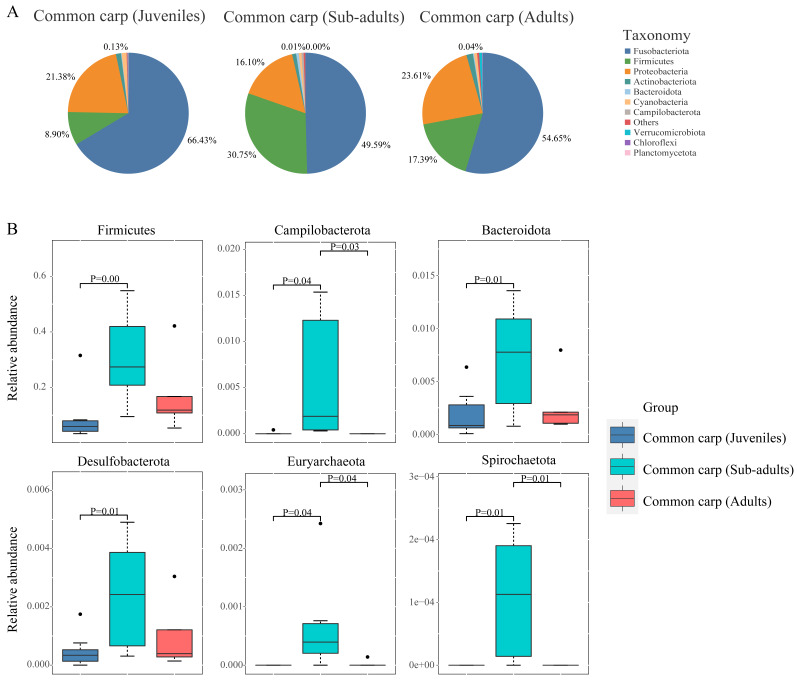
Gut microbial composition of the common carp during three different developmental stages (juvenile, sub-adult, and adult). (**A**) Pie chart for the relative abundance of the top 10 bacterial phyla in GI tracts of common carp during three different developmental stages, (**B**) Box-and-whisker plots for the relative abundance of six bacterial phyla with significant differences (*p* < 0.05) across the three developmental stages.

Throughout the period of common carp growth and development, the dominant phyla were Fusobacteria, Proteobacteria, and Firmicutes, accounting for more than 90% of the entire intestinal bacterial phylum, which is the core microbiota of common carp (Figure 3A and Table S6). The common carp harbored a similar bacterial composition at the phylum level across the three different developmental stages (Figure 3B and Table S6). We also found significant variations in the relative abundance of Firmicutes between the three stages. The relative abundance of Firmicutes was significantly higher in sub-adult (30.75 ± 6.89%) than in juveniles (8.90 ± 3.29%, MetaStats test: *p* < 0.01, Figure 3B). In addition, the other relatively low-abundance phyla (Campilobacterota, Bacteroidota, Desulfobacterota, Euryarchaeota, and Spirochaetota) were significantly different among the three stages (Figure 3B).

At the genus level, *Cetobacterium*, *Romboutsia*, and *Aeromonas* were the three dominant bacterial genera throughout the three developmental stages, and their combined relative abundances accounted for 68.91%, 75.88%, and 80.84% in the three stages, respectively. *Cetobacterium* (phylum: Fusobacteria) was the most abundant genus across the three developmental stages (Table S7). The *Romboutsia* (phylum: Firmicutes) was detected at significantly higher relative abundances in sub-adult (15.03 ± 5.51%) than in the other two stages (Juvenile:0.82 ± 0.35%, adult:1.74 ± 0.55%) (Table S7). *Clostridium sensu stricto 1* was also significantly enriched in the subadults (Table S7). Notably, *Phyllobacterium* was detected only in juveniles (Table S7).

Based on LEfSe analysis, 13 taxa with discrepancies in relative abundance were identified with an LDA score > 4 (Figure S3). The bacterial taxa from Firmicutes were the top biomarkers distinguishing the sub-adults from the other two groups. The bacteria with significant differential abundance signatures were Pseudomonadales and *Breznakia* in adults.

#### 3.3.2. Alpha and Beta Diversity of Gut Microbiota during Three Different Developmental Stages

**Figure 4:**
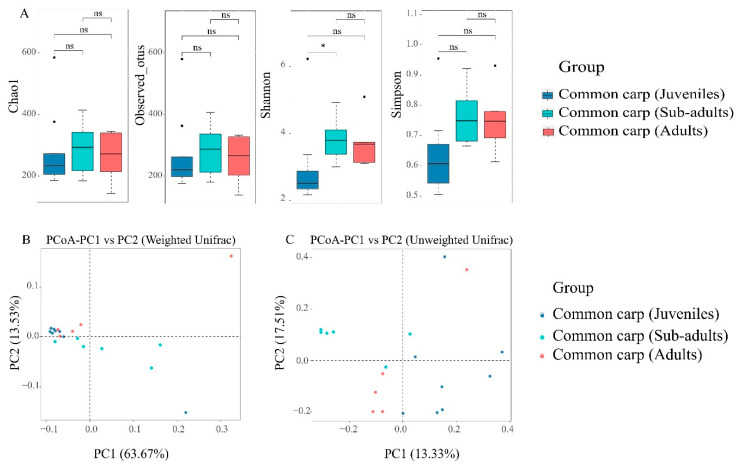
The alpha and beta diversity of gut microbial communities in common carp. (**A**) Box-and-whisker plots for comparison of alpha diversity of gut microbial communities in common carp between the three developmental stages. “*” indicates there are significant differences (*p* < 0.05) between the groups and “ns” means no significant differences. (**B**) Two-dimensional principal coordinate analysis (PCoA) of gut microbial communities between the three host’s developmental stages based on weighted UniFrac (**B**) and unweighted UniFrac (**C**) distances. Each symbol corresponds to one sample. The first two principal coordinate (PC) axes are shown.

There are no significant variations in the four alpha indices of gut microbial communities (Chao1, Shannon, Simpson, and Observed OTUs) between the three developmental stages besides the Shannon index between Juvenile and sub-adult (*p* = 0.04) (Figure 4A, and Table S8). The intestinal microbial community seems to be stable across host development.

To verify whether there were dissimilarities among the three stages, principal coordinate analysis (PCoA) was performed for each sample. Based on weighted UniFrac distances, no clear clustering pattern was observed among the three developmental stages; however, there were dissimilarities between groups despite the overlapping microbiota occurring between juveniles and adults (Figure 4B). Analysis of similarity (ANOSIM) confirmed significant differences in the bacterial community structure between juveniles and subadults (R = 0.318, *p* = 0.035) (Table 1). There was no significant difference in the microbial community structure between juveniles and adults (R = 0.213, *p* = 0.085) and between subadults and adults (R = 0.072, *p* = 0.184) under the weighted UniFrac distances (Table 1). A significant effect of development was observed on microbial community membership (unweighted UniFrac distances). The microbial communities of each group were clustered relatively closely and separated from each other (Figure 4C). This weak clustering pattern in the three microbial communities revealed a degree of intersample variability. ANOSIM confirmed significant differences in the bacterial community composition between the sub-adults and the other two stages (R > 0.283, *p* < 0.02; Table 1). We also observed extensive interindividual variation in the intestinal microbial community at each developmental stage (Figure 4).

### 3.4. Functional Potential of Bacterial Community

**Figure 5:**
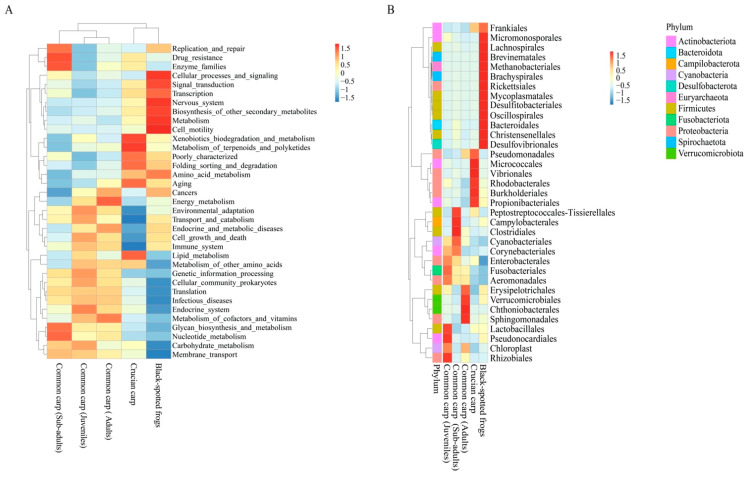
Heat map of the relative abundance of the top 35 Level II Kyoto Encyclopedia of Genes and Genomes (KEGG) pathway categories (**A**) and top 35 bacterial families (**B**) during three developmental stages in common carp and crucian carp and black-spotted frogs showing significant differences.

To further explore the relationship between the gut microbes and the host, Tax4Fun was used to predict the bacterial functional potential in all samples based on the KEGG database. The clustering heat map based on the relative abundance of the top 35 level II KEGG pathways showed that the metabolic pathways differed among the different species (Figure 5A), which was associated with the differences in core microbial families (Figure 5B). The black-spotted frogs were enriched in functional categories associated with metabolism, cell motility, biosynthesis of other metabolites, nervous system, transcription, cellular processes, and signaling. Functional partitioning was also found for level II KEGG pathway categories between the sub-adult common carp and sub-adult crucian carp. Functional categories for carbohydrate metabolism, glycan biosynthesis and metabolism, nucleotide metabolism, metabolism of cofactors and vitamins, and membrane transport were more abundant in common carp across life stages (Figure 5A). The heatmap of the dominant functional categories, divided by developmental stages in common carp, was also consistent with the microbial composition changes across life stages. We found that bacteria associated with energy metabolism, lipid metabolism, and endocrine and metabolic diseases were more abundant in the adult common carp.

## 4. Discussion

There has been increasing interest in the gut microbiota of commercial and wild fish because of their close association with health, metabolism, adaptability, and development. Rice fish are widely raised in central and southern China, owing to their considerable economic and ecological value. In previous studies, the gut microbiota in commercial fish from monoculture ponds and the rice-fish coculture system were compared [21,66]. Moreover, the effects of diet on the gut microbiota, intestinal structure, and immunity of rice fishes have been investigated in common carp [12] and rice field eel [67]. However, little attention has been focused on the temporal pattern of gut microbial communities in rice fish during their different developmental stages in RFS. In this study, we characterized the gut microbial communities of an aquaculture fish species (common carp) at various stages of development in the RFS of China and compared the differences in gut microflora between sympatric aquatic animals (common carp, crucian carp, and black-spotted frogs).

Previous studies have indicated that the gut microbiota of various commercial fish (such as silver carp, bighead carp, grass carp, and banded catfish) is dominated by the phyla Firmicutes and Bacteroidetes, with a combined relative abundance of >80% for these three phyla [22,68]. Consistent with previous results, the phyla Firmicutes, Proteobacteria, and Fusobacteria also dominated the gut microbiota of the common carp and crucian carp (Figure 1A and Table S3) in our dataset, suggesting that the gastrointestinal tracts of rice fishes harbored a relatively conserved microbial consortium. The black-spotted frog, common carp, and crucian carp coexist in rice paddy fields, prey on insect pests, and are regarded as beneficial in rice fields [51]. Even so, it was evident that distinct gut microbiota were found in the three aquatic species (Figure 1A, Figure 2B, and Table 1) due to the potentially separated dietary niche and further phylogenetic relationships. Meanwhile, it is possible that the black-spotted frog may obtain distinctive microbiota from the environment outside the rice fields. Firmicutes and Euryarchaeota were the dominant phyla in black-spotted frogs (Figure 1A and Table S3), in accordance with previous studies [69,70]. Firmicutes have been reported to decompose carbohydrates and plant cell wall components, and produce short-chain fatty acids as byproducts of fermentation [71,72]. The higher relative abundance of Firmicutes in the intestines of frogs may be linked to higher efficiency of energy harvesting from their diet due to the abundant food present in farmland environments [73]. Compared to artificial feeding, rice fish raised in paddy fields also harbored more abundant Firmicutes, with higher Firmicutes/Bacteroidota ratios (Table S9). A higher Firmicutes/Bacteroidetes ratio suggests a higher efficiency of energy uptake from food [74]. The higher abundance of Firmicutes in rice fish may facilitate the acquisition of energy and may contribute to an increase in fat accumulation. This is because the rice fish faced limited food in their natural diets, in contrast to the abundant food in artificial feeding. Previous studies have demonstrated that diet is an important factor influencing the intestinal microflora of fish [75,76,77]. Carnivorous fishes harbor a relatively higher abundance of Fusobacteria, while the relative abundance of Firmicutes phylum is low [78]. A high relative abundance of Firmicutes and a low relative abundance of Fusobacteria were observed in herbivorous fish [79]. Members of the phylum Fusobacteria have been reported to ferment amino acids and peptides to produce various organic acids [14,80,81]. The high proportion of Fusobacteria in the two carp species may be an adaptation to a high-protein diet in our study (Figure 1 and Table S3), which is also correlated with the enriched functional categories associated with the metabolism of other amino acids, metabolism of cofactors and vitamins, and membrane transport in carp (Figure 5). Common carp mainly prey on insects or benthic worms in paddy fields [33]. In contrast, crucian carp are omnivorous and forage on planktonic algae and animals [82], which could explain the higher abundance of Fusobacteria in common carp than in crucian carp (Figure 1A and Table S3). The phylum Proteobacteria was associated with a variety of metabolic functions [83] and included various potential pathogenic genera (such as *Pseudomonas* and *Aeromonas*), these two pathogenic genera were also detected in relatively higher proportions in GI tracts of common carp and crucian carp (Table S4).

Although water has typically been found to contribute to the fish gut microbiota [84,85], we detected differences in gut microbial composition (Figure 1A), alpha diversity (Figure 2A), and community structure (Figure 2B) between water samples and three aquatic animals. In aquaculture systems, the aquatic environment microbiota is assumed to be one of the main sources of fish microbiota [86,87,88]. In this study, we found that the gut microbial communities of fishes were significantly different from those of the water environment (Figure 1A and Table S3), consistent with previous studies that demonstrated that the gut microbial community of fishes is not a simple reflection of the environmental microbial community [89,90,91]. In addition, different carp were reared under the same environmental conditions, and the gut microbial composition (Figure 1) and community structure (Figure 2B) were different between these two carp species, despite some overlap of microbial communities between common carp and crucian carp in the PCoA analysis (Figure 4B). Host genetics may play an important role in shaping the gut microbiome of the two carp species, despite having a close genetic relationship. The differences in gut microbiota may also result from species-specific diet, gut morphology, and phylogeny, as demonstrated in previous studies [29,92,93].

Host developmental processes have significant effects on fish gut microbiota [44,94]. Stage-specific signatures of gut microbiota have been extensively investigated in a model fish, zebrafish, at seven time points during development [44,94]. In this study, the gut microbiota of common carp assembled into distinct communities at different stages during host development (Figure 4B and Table S4), suggesting a correlation between development and gut microbial communities. Extensive interindividual variation was observed during different developmental stages, which is also the case in other fish hosts [44,92]. But present study indicated that no significant differences were found in alpha diversity among all developmental stages of rice fish (Figure 4A and Table S8). A stable water environment and food resources could explain the non-significant changes in alpha diversity across the ontogeny in the short coculture of common carp. Most of the microbial diversity could be summarized into three significant phyla: Fusobacteria, Proteobacteria, and Firmicutes, throughout the developmental stages of common carp (Table S6 and Figure S4A). Despite the observed compositional fluctuations in some bacterial taxa during the three developmental stages (Figure 3B and Figure 4), these three phyla constituted the core microbiota in the gut of the common carp (Figure 3A), indicating that common carp might be selective for the colonization of dominant intestinal bacteria. Similarly, a previous study showed that the relative abundance (evenness) of different bacterial classes did not change with age in zebrafish [44]. However, previous studies have demonstrated that gut bacterial communities associated with developmental stages in fish vary widely in diversity and abundance [95,96,97].

Differences in the relative abundance of bacterial phyla between the three developmental stages were observed, with Firmicutes, Campilobacterota, Bacteroidota, Desulfobacterota, Euryarchaeota, and Spirochaetota being higher in sub-adults than in juveniles and adults (Figure 3 and Table S6). Firmicutes play a key role in the degradation of carbohydrates and plant cell wall components into volatile fatty acids that provide energy to the hosts [71,98,99]. Therefore, a higher abundance of Firmicutes in the gut microbiota of sub-adults may be associated with their increased foraging on plants (e.g., weeds and phytoplankton) and can facilitate nutrient absorption by the host [72,100] and contribute to reducing weeds in RSF. This was also supported by the fact that functional categories for glycan biosynthesis and metabolism, carbohydrate metabolism, and enzyme families were more abundant in the subadult group (Figure 5). The genus *Cetobacterium* within the phylum Fusobacteriota, known to be positively associated with protein and polypeptide digestion, is beneficial to the host by producing vitamin B12 and butyrate and their antibacterial properties [101], which is the most abundant genus in a variety of freshwater fish species [90]. Therefore, *Cetobacterium* species are considered indicators of healthy fish [89,102,103]. The relatively high abundance of *Cetobacterium* in all three developmental stages of the common carp could be explained by their high-protein diets. Common carp prey on insect pests during their life stages in the RSF. The *Romboutsia* and *Clostridium sensu stricto I* genera were the two most abundant genera within the phylum Firmicutes in the GI tracts of common carp (Table S7). *Romboutsia* species are capable of utilizing carbohydrates, can ferment various amino acids [104,105,106], and are also involved in the regulation of lipid metabolism [107,108], which is also regarded as a probiotic to promote host growth and resistance to pathogens in the fish intestine [66]. *Romboutsia* exhibited a higher relative abundance in sub-adults (Table S8). Increased intake of high-protein diets during summer may have resulted in a higher relative abundance of *Romboutsia* in common carp. The genus *Aeromonas* within Proteobacteria was detected at a relatively high relative abundance (11.54–13.64%) across host development (Table S7). *Aeromonas* can enhance the digestive function of the host in the intestine of healthy fish [41]. However, some members of the genus *Aeromonas*, such as *Aeromonas hydrophila*, are conditionally pathogenic bacteria [109] that cause an inflammatory response and gut disturbances in fish [93]. This finding is consistent with previous studies showing that the fish gut harbors opportunistic pathogens [110], which should be considered in aquaculture.

## 5. Conclusions

This study analyzed and compared characteristics of the composition and diversity of the intestinal microbial communities of two commercial carp (common carp and crucian carp) and a sympatric frog (black-spotted frog) in RFS. The phyla Firmicutes, Proteobacteria, and Fusobacteria, dominated the gut microbiota of the common carp and crucian carp. We found that the gut microbial composition and community structure differed between three sympatric aquatic species in RFS. Specifically, there was a stable core group of microbiota in common carp during host development, with a small variation in alpha diversity. The gut microbial communities of common carp also underwent changes in beta diversity across the three developmental stages, with an increase in the relative abundance of Firmicutes in sub-adults. Our results may increase the understanding of gut microbial community composition and diversity in commercial fish in RFS and improve existing strategies in the aquaculture of traditional rice fishes.

## Figures and Tables

**Table 1:** Results of ANOSIM tests with 999 permutations on the gut microbial community between three sympatric aquatic species and water environment collected in July and between the three developmental stages in common carp.

Pair-Wised Comparison	Weighted Unifrac		Unweighted Unifrac	
	R Value	*p* Value	R Value	*p* Value
Common carp—Crucian carp	0.284	0.159	0.617	0.015
Common carp—Black-spotted frogs	0.737	0.005	0.372	0.005
Crucian carp—Black-spotted frogs	0.574	0.005	0.648	0.015
Black-spotted frogs—Water	0.679	0.010	1	0.030
Crucian carp—Water	1	0.109	1	0.075
Common carp—Water	0.988	0.020	1	0.025
Common carp (Juvenile)—Common carp (Sub-adult)	0.318	0.035	0.442	0.005
Common carp (Juvenile)—Common carp (Adult)	0.213	0.085	0.204	0.075
Common carp (Sub-adult)—Common carp (Adult)	0.072	0.184	0.283	0.020

Note: R values > 0 indicates the difference between groups is greater than that within groups. *p* < 0.05 indicates significant differences between groups.

## Data Availability

The raw sequence data reported in this paper have been deposited in the Genome Sequence Archive (Genomics, Proteomics, and Bioinformatics 2017) at the National Genomics Data Center (Nucleic Acids Research 2021), China National Center for Bioinformation/Beijing Institute of Genomics, Chinese Academy of Sciences, under accession number CRA006855 that are publicly accessible at https://bigd.big.ac.cn/gsa, accessed on 9 May 2022.

## Supplementary Materials

The following supporting information can be downloaded at: https://www.mdpi.com/article/10.3390/microorganisms10122373/s1, Figure S1. A Venn diagram showing the overlapping number of ASV between the three developmental stages in the common carp. B Venn diagram showing the overlapping number of ASV between the three developmental stages in the common carp during three developmental stages and sub-adult crucian carp (July). C Rarefaction curves of species richness of gut microbiota in common carp, crucian carp, black-spotted frog and water environment in RFS. Figure S2. Bacterial taxa significantly differentiated between three sympatric aquatic species, as determined by LEfSe. Linear discriminant analysis (LDA) scores were interpreted as the degree of difference in relative abundance. Figure S3. Bacterial taxa significantly differentiated between three developmental stages in common carp, as determined by LEfSe. Table S1. The number of bacterial taxa at various taxonomic levels was identified in each group. Table S2. Alpha diversity indices (Goods coverage, Chao1, Observed ASV, Shannon, and Simpson; mean ± SE) between common carp, crucian carp, black-spotted frogs, and water collected in July. Table S3. Pairwise comparison of the average relative abundance ± standard error (SE) (%) of the top 10 bacterial phyla between the four groups in July. Table S4. Pairwise comparison of average relative abundance ± standard error (SE) (%) of the top 20 bacterial genera between the three species and water in July. Table S5. Summary and pairwise comparison of alpha diversity estimators (Chao1, observed ASV, Shannon, and Simpson) for gut microbial communities between the four groups collected in July based on the Wilcoxon rank-sum test. Table S6. Pairwise comparison of the average relative abundance ± standard error (SE) (%) of the top 10 bacterial phyla between the three developmental stages in common carp. Table S7. Pairwise comparison of the average relative abundance ± standard error (SE) (%) of the top 20 bacterial genera between the three developmental stages in common carp. Table S8. Summary and pairwise comparison of alpha diversity estimators (Chao1, observed ASV, Shannon, and Simpson) for gut microbial communities between the three different developmental stages in common carp based on the Wilcoxon rank-sum test. Note: Different letters indicate differences between seasons (*p* < 0.05). SE, standard error. Table S9. Composition of the dominant microbial phyla in the GI tracts of aquatic animals in the rice-fish coculture system and raised under artificial feeding based on previous studies.
