# Supplementary material for: Characterization and Dynamics of the Gut Microbiota in Rice Fishes at Different Developmental Stages in Rice-Fish Coculture Systems
Source: Microorganisms. 2022 Nov 30;10(12):2373. doi: 10.3390/microorganisms10122373 (PMC9787495; doi:10.3390/microorganisms10122373)
Supplement: Supplementary file 1 [file microorganisms-10-02373-s001.zip › Supplementary Table S1.pdf]

Supplementary Table S1. The number of bacterial taxa at various taxonomic levels identified in each group.

Group	ASV	Phylum	Class	Order	Family	Genus	Species
Common carp (Juvenile)	1242	20	39	98	170	311	147
Common carp (sub-adult)	1017	20	35	101	170	299	140
Common carp (adult)	777	16	38	92	145	241	87
Crucian carp (July)	923	22	41	111	185	308	140
Black-spotted frogs (July)	1298	22	41	103	179	336	165
Water (July)	1617	27	51	131	223	380	158
