# Supplementary material for: Characterization and Dynamics of the Gut Microbiota in Rice Fishes at Different Developmental Stages in Rice-Fish Coculture Systems
Source: Microorganisms. 2022 Nov 30;10(12):2373. doi: 10.3390/microorganisms10122373 (PMC9787495; doi:10.3390/microorganisms10122373)
Supplement: Supplementary file 1 [file microorganisms-10-02373-s001.zip › Supplementary Table S2.pdf]

Supplementary Table S2. Alpha diversity indices (Goods coverage, Chao1, Observed ASV, Shannon, and Simpson; mean ± SE) between common carp, crucian carp, black-spotted frogs, and water collected in July.

Group	Richness estimates			Diversity estimates	
	Goods coverage	Chao1 (Mean±SE)	Observed ASV (Mean±SE)	Shannon (Mean±SE)	Simpson (Mean±SE)
Common carp (*Cyprinus carpio*)	0.9996	278.22±24.74	273.47±24.75	3.47±0.25	0.71±0.03
Crucian carp (*Carassius auratus*)	0.9997	420.60±29.69	414.33±28.67	4.72±0.13	0.86±0.02
Black-spotted frogs (*Pelophylax nigromaculatus*)	1.0000	375.44±43.28	373.67±43.10	5.31±0.41	0.91±0.27
Water	0.9992	1139.95±145.51	1132.50±142.36	8.31±0.15	0.99±0.00
