# Supplementary material for: Characterization and Dynamics of the Gut Microbiota in Rice Fishes at Different Developmental Stages in Rice-Fish Coculture Systems
Source: Microorganisms. 2022 Nov 30;10(12):2373. doi: 10.3390/microorganisms10122373 (PMC9787495; doi:10.3390/microorganisms10122373)
Supplement: Supplementary file 1 [file microorganisms-10-02373-s001.zip › Supplementary Table S3.pdf]

Supplementary Table S3. Pairwise comparison of the average relative abundance ± standard error (SE) (%) of the top 10 bacterial phyla between the four groups in July. Different superscript letters indicate differences between groups ($P < 0.05$).

Phylum level	Common carp	Crucian carp	Black-spotted frogs	Water
	(Mean±SE)	(Mean±SE)	(Mean±SE)	(Mean±SE)
Firmicutes	30.75% ± 6.89%^{a}	32.19% ±15.08%^{a}	70.39% ± 6.77%^{b}	6.17% ± 3.38%^{c}
Fusobacteriota	49.59% ± 11.24%^{a}	19.20% ± 7.01%^{b}	1.24% ± 0.36%^{c}	0.29% ± 0.05%^{d}
Proteobacteria	16.10% ± 6.05%^{a}	33.08% ± 13.32%^{a}	6.46% ± 1.56%^{a}	57.35% ± 1.12%^{b}
Euryarchaeota	0.07% ± 0.04%^{a}	0.02% ± 0.01%^{a}	10.07% ± 5.37%^{b}	0^{ab}
Actinobacteriota	1.07% ± 0.41%^{a}	13.52% ± 11.13%^{a}	2.37% ± 0.90%^{a}	13.23% ± 1.51%^{b}
Cyanobacteria	0.71% ± 0.40%^{a}	0.55% ± 0.14%^{a}	0.52% ± 0.23%^{a}	7.17% ± 2.68%^{b}
Bacteroidota	0.72% ± 0.21%^{a}	0.73% ± 0.15%^{a}	4.56% ± 3.85%^{ab}	11.49% ± 0.65%^{b}
Spirochaetota	0.01% ± 0.00%^{a}	0.01% ± 0.00%^{a}	1.23% ± 1.12%^{ab}	0.19% ± 0.05%^{b}
Desulfobacterota	0.24% ± 0.08%^{a}	0.23% ± 0.18%^{a}	2.15% ± 0.82%^{b}	0.11% ± 0.03%^{a}
Verrucomicrobiota	0.03% ± 0.07%^{a}	0.03% ± 0.00%^{a}	0.09% ± 0.06%^{ab}	0.90% ± 0.05%^{b}
others	0.72% ± 0.29%^{a}	0.43% ± 0.11%^{a}	0.92% ± 0.38%^{a}	3.10% ± 0.40%^{b}
