# Supplementary material for: Characterization and Dynamics of the Gut Microbiota in Rice Fishes at Different Developmental Stages in Rice-Fish Coculture Systems
Source: Microorganisms. 2022 Nov 30;10(12):2373. doi: 10.3390/microorganisms10122373 (PMC9787495; doi:10.3390/microorganisms10122373)
Supplement: Supplementary file 1 [file microorganisms-10-02373-s001.zip › Supplementary Table S4.pdf]

Supplementary Table S4. Pairwise comparison of average relative abundance ± standard error (SE) (%) of the top 20 bacterial genera between the three species and water in July. Different superscript letters indicate differences between groups ($P < 0.05$).

Phylum level	Genus level	Common carp	Crucian carp	Black-spotted frogs	Water
		Mean ± SE	Mean ± SE	Mean ± SE	Mean ± SE
Fusobacteriota	*Cetobacterium*	49.24 ± 11.20%^{a}	17.95 ± 6.91%^{b}	0.86 ± 0.38%^{c}	0.07 ± 0.07%^{c}
Firmicutes	*Romboutsia*	15.00 ± 5.50%^{a}	15.70 ± 12.50%^{a}	0.47 ± 0.17%^{b}	0.53 ± 0.33%^{b}
	TC1	0.96 ± 0.36%^{a}	1.11 ± 0.70%^{a}	22.68 ± 5.47%^{b}	0.18 ± 0.18%^{a}
Proteobacteria	*Aeromonas*	11.51 ± 5.52%^{a}	3.00 ± 1.44%^{ab}	0.26 ± 0.04%^{b}	0.27 ± 0.11%^{b}
	Vibrio	0.37 ± 0.16%^{a}	10.11 ± 9.75%^{a}	0.27 ± 0.03%^{a}	0.15 ± 0.15%^{a}
	Pseudomonas	0.44 ± 0.15%^{a}	11.80 ± 7.10%^{a}	0.46 ± 0.16%^{a}	1.19 ± 0.20%^{b}
Firmicutes	*Mycoplasma*	0.03 ± 0.02%^{a}	0.03 ± 0.03%^{a}	4.04 ± 3.16%^{a}	< 0.01^{a}
Actinobacteriota	*Aurantimicrobium*	0.04 ± 0.03%^{a}	6.50 ± 5.98%^{a}	0.02 ± 0.02%^{ab}	0.91 ± 0.09%^{c}
Bacteroidota	*Parabacteroides*	0.02 ± 0.01%^{a}	0.03 ± 0.03%^{a}	2.38 ± 2.25%^{a}	0.01 ± 0.01%^{a}
Firmicutes	*ZOR0006*	0.81 ± 0.65%^{a}	1.21 ± 0.61%^{a}	0.01 ± 0.00%^{a}	0.02 ± 0.02%^{a}

Proteobacteria	*Hydrogenophaga*	0^{a}	0.12 ± 0.01%^{b}	0.03 ± 0.03%^{ab}	8.41 ± 2.47%^{c}
Firmicutes	*Anaerorhabdus_furcosa_group*	0.11 ± 0.11%^{a}	0.01 ± 0.01%^{a}	0.07 ± 0.05%^{a}	< 0.01^{a}
	Clostridium_sensu_stricto_1	4.30 ± 1.24%^{a}	2.62 ± 1.93%^{ab}	0.97 ± 0.54%^{b}	0.38 ± 0.24%^{b}
Actinobacteriota	*hgcI_clade*	0^{a}	0.30 ± 0.16%^{b}	0.12 ± 0.12%^{ab}	7.17 ± 0.97%^{c}
	Breznakia	0.02 ± 0.01%^{a}	0.08 ± 0.05%^{a}	0.04 ± 0.02%^{a}	0^{b}
Firmicutes	*Proteocatella*	0.06 ± 0.04%^{a}	0.28 ± 0.23%^{a}	< 0.01^{a}	0^{a}
	Paraclostridium	1.47 ± 0.52%^{a}	0.90 ± 0.50%^{a}	2.53 ± 1.13%^{a}	0.05 ± 0.05%^{b}
	C39	0^{a}	0.12 ± 0.06%^{b}	0.04 ± 0.04%^{ab}	6.37 ± 0.38%^{c}
Proteobacteria	*Escherichia-Shigella*	0.15 ± 0.06%^{a}	0.26 ± 0.15%^{a}	0.05 ± 0.05%^{a}	0.44 ± 0.43%^{a}
	Polynucleobacter	0^{a}	0.15 ± 0.08%^{b}	0.03 ± 0.03%^{ab}	5.17 ± 0.26%^{c}
	Others	15.47 ± 2.38%	27.86 ± 8.04%	64.71 ± 6.08%	73.84 ± 1.73%
