# Supplementary material for: Characterization and Dynamics of the Gut Microbiota in Rice Fishes at Different Developmental Stages in Rice-Fish Coculture Systems
Source: Microorganisms. 2022 Nov 30;10(12):2373. doi: 10.3390/microorganisms10122373 (PMC9787495; doi:10.3390/microorganisms10122373)
Supplement: Supplementary file 1 [file microorganisms-10-02373-s001.zip › Supplementary Table S5.pdf]

Supplementary Table S5. Summary and pairwise comparison of alpha diversity estimators (Chao1, observed ASV, Shannon, and Simpson) for gut microbial communities between the four groups collected in July based on the Wilcoxon rank-sum test. Note: different letters indicate differences between seasons ($p < 0.05$). SE, standard error.

Group	Richness estimates		Diversity estimates	
	Chao1 (Mean±SE)	Observed ASV (Mean±SE)	Shannon (Mean±SE)	Simpson (Mean±SE)
Common carp	285.50±36.50[a]	285.00±35.59[a]	3.80±0.29[a]	0.76±0.04[a]
Crucian carp	401.04±48.58[b]	401.00±28.02[b]	4.73±0.13[abc]	0.86±0.02[abc]
Black-spotted frogs	371.31±41.86[a]	371.00±42.00[a]	5.30±0.41[b]	0.91±0.27[b]
Water	847.03±57.65[c]	847.00±57.66[c]	8.01±0.02[c]	0.99±0.00[c]
