# Supplementary material for: Characterization and Dynamics of the Gut Microbiota in Rice Fishes at Different Developmental Stages in Rice-Fish Coculture Systems
Source: Microorganisms. 2022 Nov 30;10(12):2373. doi: 10.3390/microorganisms10122373 (PMC9787495; doi:10.3390/microorganisms10122373)
Supplement: Supplementary file 1 [file microorganisms-10-02373-s001.zip › Supplementary Table S6.pdf]

Supplementary Table S6. Pairwise comparison of the average relative abundance ± standard error (SE) (%) of the top 10 bacterial phyla between the three developmental stages in common carp. Different superscript letters indicate differences between groups (P < 0.05).

Phylum level	Common carp (Juveniles)	Common carp (Sub-adults)	Common carp (Adults)
	(Mean ± SE)	(Mean ± SE)	(Mean ± SE)
Fusobacteriota	66.43 ± 7.48%[a]	49.59 ±11.24%[a]	54.65 ± 13.07%[a]
Firmicutes	8.90 ± 3.29%[a]	30.75 ± 6.89%[b]	17.39 ± 6.45%[ab]
Proteobacteria	21.38 ± 2.62%[a]	16.10 ± 6.05%[a]	23.61 ± 4.36%[a]
Actinobacteriota	1.36 ± 1.13%[a]	1.07 ± 0.41%[a]	0.82 ± 0.60%[a]
Cyanobacteria	1.29 ± 0.84%[a]	0.71 ± 0.40%[a]	1.76 ± 1.56%[a]
Verrucomicrobiota	0.06 ± 0.04%[a]	0.03 ± 0.07%[a]	0.76 ± 0.62%[a]
Campilobacterota	0.01 ± 0.01%[a]	0.59 ± 0.30%[b]	0[a]
Bacteroidota	0.19 ± 0.08%[a]	0.72 ± 0.21%[b]	0.28 ± 0.13%[ab]

Planctomycetota	0.13 ± 0.11%[a]	0[a]	0.04 ± 0.02%[a]
Chloroflexi	0.04 ± 0.04%[a]	0.01 ± 0.01%[a]	0.13 ± 0.13%[a]
others	0.21 ± 0.14%[a]	0.44 ± 0.10%[a]	0.56 ± 0.46%[a]
