# Supplementary material for: Characterization and Dynamics of the Gut Microbiota in Rice Fishes at Different Developmental Stages in Rice-Fish Coculture Systems
Source: Microorganisms. 2022 Nov 30;10(12):2373. doi: 10.3390/microorganisms10122373 (PMC9787495; doi:10.3390/microorganisms10122373)
Supplement: Supplementary file 1 [file microorganisms-10-02373-s001.zip › Supplementary Table S7.pdf]

Supplementary Table S7. Pairwise comparison of average relative abundance ± SE (Standard Error) (%) of the top 20 bacterial genera between the three developmental stages in common carp. Different superscript letters indicate a difference between groups ($P < 0.05$).

Phylum level	Genus level	Common carp (Juveniles)	Common carp (Sub-adults)	Common carp (Adults)
		Mean±SE	Mean±SE	Mean±SE
Fusobacteriota	*Cetobacterium*	66.40 ± 7.47%^{a}	49.32 ± 11.22%^{a}	53.53 ± 12.80%^{a}
Firmicutes	*Romboutsia*	0.82 ± 0.35%^{a}	15.03 ± 5.51%^{b}	1.74 ± 0.55%^{a}
Proteobacteria	*Aeromonas*	13.62 ± 2.94%^{a}	11.54 ± 5.53%^{a}	13.64 ± 4.23%^{a}
	Pseudomonas	0.07 ± 0.03%^{a}	0.44 ± 0.15%^{b}	4.36 ± 3.67%^{ab}
Firmicutes	*ZOR0006*	2.04 ± 1.66%^{a}	0.81 ± 0.64%^{a}	1.35 ± 0.71%^{a}
	Anaerorhabdus furcosus	0^{a}	0.12 ± 0.11%^{a}	2.19 ± 2.08%^{a}
	Clostridium_sensu_stricto_I	1.11 ± 0.25%^{a}	4.30 ± 1.24%^{b}	2.80 ± 1.01%^{ab}
	Breznakia	< 0.01^{a}	0.01 ± 0.01%^{a}	3.00 ± 1.81%^{a}
Proteobacteria	*Phyllobacterium*	1.03 ± 1.00%^{a}	0^{a}	0^{a}
Firmicutes	*Proteocatella*	< 0.01^{a}	0.06 ± 0.04%^{a}	1.44 ± 1.40%^{a}
	Enterococcus	0.93 ± 0.63%^{a}	0.06 ± 0.03%^{a}	0.10 ± 0.06%^{a}
Proteobacteria	*Bosea*	0.56 ± 0.51%^{a}	0.01 ± 0.01%^{a}	0.04 ± 0.03%^{a}
Firmicutes	*Paraclostridium*	0^{a}	1.47 ± 0.52%^{b}	0.13 ± 0.06%^{c}

Proteobacteria	*Methylobacterium-Methylorubrum*	0.49 ± 0.46%[a]	0.01 ± 0.00%[a]	0.37 ± 0.32%[a]
	Escherichia-Shigella	0.68 ± 0.35%[a]	0.15 ± 0.06%[a]	0.04 ± 0.03%[b]
Firmicutes	*Candidatus_Arthromitus*	0.13 ± 0.10%[a]	1.01 ± 0.48%[b]	0[a]
Proteobacteria	*Allorhizobium*	0.41 ± 0.35%[a]	0.01 ± 0.01%[a]	0.46 ± 0.42%[a]
Fusobacteriota	*Fusobacterium*	0.02 ± 0.01%[a]	0.27 ± 0.14%[b]	1.10 ± 0.54%[b]
Proteobacteria	*Plesiomonas*	0.12 ± 0.04%[a]	0.96 ± 0.40%[b]	0.96 ± 0.31%[b]
Firmicutes	*TC1*	0[a]	0.96 ± 0.36%[b]	0[a]
	Others	11.57 ± 5.34%	13.49 ± 2.10%	13.20 ± 6.65%
