# Supplementary material for: Characterization and Dynamics of the Gut Microbiota in Rice Fishes at Different Developmental Stages in Rice-Fish Coculture Systems
Source: Microorganisms. 2022 Nov 30;10(12):2373. doi: 10.3390/microorganisms10122373 (PMC9787495; doi:10.3390/microorganisms10122373)
Supplement: Supplementary file 1 [file microorganisms-10-02373-s001.zip › Supplementary Table S8.pdf]

Supplementary Table S8. Summary and pairwise comparison of alpha diversity estimators (Chao1, observed ASV, Shannon, and Simpson) for gut microbial communities between the three different developmental stages in common carp based on the Wilcoxon rank-sum test. Note: Different letters indicate differences between seasons ($P < 0.05$). SE, standard error.

Group	Richness estimates		Diversity estimates	
	**Chao1 (Mean±SE)**	**Observed ASV (Mean±SE)**	**Shannon (Mean±SE)**	**Simpson (Mean±SE)**
Common carp (Juveniles)	277.98 ± 47.99^{a}	272.50 ± 48.41^{a}	3.02 ± 0.48^{a}	0.64 ± 0.05^{a}
Common carp (Sub-adults)	284.68 ± 36.45^{a}	283.50 ± 36.15^{a}	3.83 ± 0.28^{b}	0.76 ± 0.04^{a}
Common carp (Adults)	258.38 ± 38.26^{a}	253.60 ± 37.11^{a}	3.75 ± 0.36^{ab}	0.75 ± 0.05^{a}
