# Supplementary material for: Characterization and Dynamics of the Gut Microbiota in Rice Fishes at Different Developmental Stages in Rice-Fish Coculture Systems
Source: Microorganisms. 2022 Nov 30;10(12):2373. doi: 10.3390/microorganisms10122373 (PMC9787495; doi:10.3390/microorganisms10122373)
Supplement: Supplementary file 1 [file microorganisms-10-02373-s001.zip › Supplementary Table S9.pdf]

Supplementary Table S9. Composition of the dominant microbial phyla in the GI tracts of aquatic animals in the rice-fish coculture system and raised under artificial feeding, based on previous studies.

Fish species	Methods	Aquaculture model	Firmicutes	Bacteroidota	Proteobacteria	Fusobacteria	References
Common carp (*Cyprinus carpio*)	16S rRNA	Rice-fishes co-culturing	30.77%	0.72%	16.16%	49.51%	This study
Crucian carp (*Carassius cuvieri*)			32.19%	0.73%	33.08%	19.20%	
Furuili 2 (*Cyprinus carpio*)	16S rRNA	Rice-fishes co-culturing	15.40%	1.56%	39.39%	38.55%	(Nei et al. 2022)
		Artificial feeding	0.51%	9.80%	21.87%	58.27%	
			0.20%	0.01%	46.63%	53.14%	
Banded Catfish (*Tachysurus fulvidraco*♀× *Pseudobagrus vachellii*♂)	16S rRNA	Rice-fishes co-culturing	36.70%	20.20%	31.20%	10.40%	(Zhu et al. 2021)
		Artificial feeding	10.70%	12.50%	64.80%	10.80%	
Silver carp (*Hypophthalmichthys molitrix*)	16S rRNA	Rice-fishes co-culturing	8.22%	8.02%	38.93%	6.46%	(Li et al. 2018)
Bighead carp (*Hypophthalmichthys nobilis*)			10.84%	7.63%	39.45%	29.31%	
Grass carp (*Ctenopharyngodon idella*)			11.19%	18.32%	37.54%	14.17%	
Common carp (*Cyprinus carpio*)			24.56%	4.23%	35.30%	19.40%	
Grass carp (*Ctenopharyngodon idellus*)	16S rRNA	Rice-fishes co-culturing	9.72%	19.03%	5.95%	64.58%	(Li et al. 2014)
Crucian carp (*Carassius cuvieri*)			0.42%	0.11%	1.81%	97.50%	
Bighead carp (*Hypophthalmichthys nobilis*)			18.93%	4.85%	16.77%	41.75%	

Common carp (*Cyprinus carpio*)	DGGE	Artificial feeding	 7.70%<br>11.11%	95.00%<br>84.62%<br>74.07%	5.00%	(Yang et al. 2019)

References:

1. Li, J.; J, Ni.; J, Li.; C, Wang.; X, Li.; S, Wu.; T, Zhang.; Y, Yu.; Q, Yan. Comparative study on gastrointestinal microbiota of eight fish species with different feeding habits, *J Appl Microbiol* **2014**.*117*, 1750-60.
2. Li, X.; Y, Yu.; C, Li.; Q, Yan. Comparative study on the gut microbiotas of four economically important Asian carp species, *Sci China Life Sci* **2018**. *61*, 696-705.
3. Yang, S.; J, Du.; J, Luo.; Y, Zhou.; Y, Long.; G, Xu.; L, Zhao.; Z, Du.; T, Yan. Effects of different diets on the intestinal microbiota and immunity of common carp (*Cyprinus carpio*). *Journal of Applied Microbiology* **2019**, *127*, 1327-38.
4. Nie, Z.J.; Xu, G.C.; Shao, N.L.; Wang, B.Z.; Gao, J.C.; Xu, P.; He, J. Comparison of gut microbiota in carps from fish monoculture ponds and the rice-fish co-culture system in Hani Terraces Acta Microbiologica Sinica. **2022**, *62*, 1473-1484. http://dx.doi.org/10.13343/j.cnki.wsxb.202104. (In Chinese)
5. Zhu, J.H.; Qiang, J.; Xu, G.C.; Tao, Y. F.; Bao, J.W.; Xu, P. Microbial community structure of hybrid yellow catfish in rice-fish co-culture system in hani terrace. *Acta Hydrobiologica Sinica* **2021**, *45*, 1232-1242. https://doi.org/10.7541/2021.2020.179. (In Chinese)
