# Supplementary figures and images for: Characterization and Dynamics of the Gut Microbiota in Rice Fishes at Different Developmental Stages in Rice-Fish Coculture Systems
Source: Microorganisms. 2022 Nov 30;10(12):2373. doi: 10.3390/microorganisms10122373 (PMC9787495; doi:10.3390/microorganisms10122373)

A

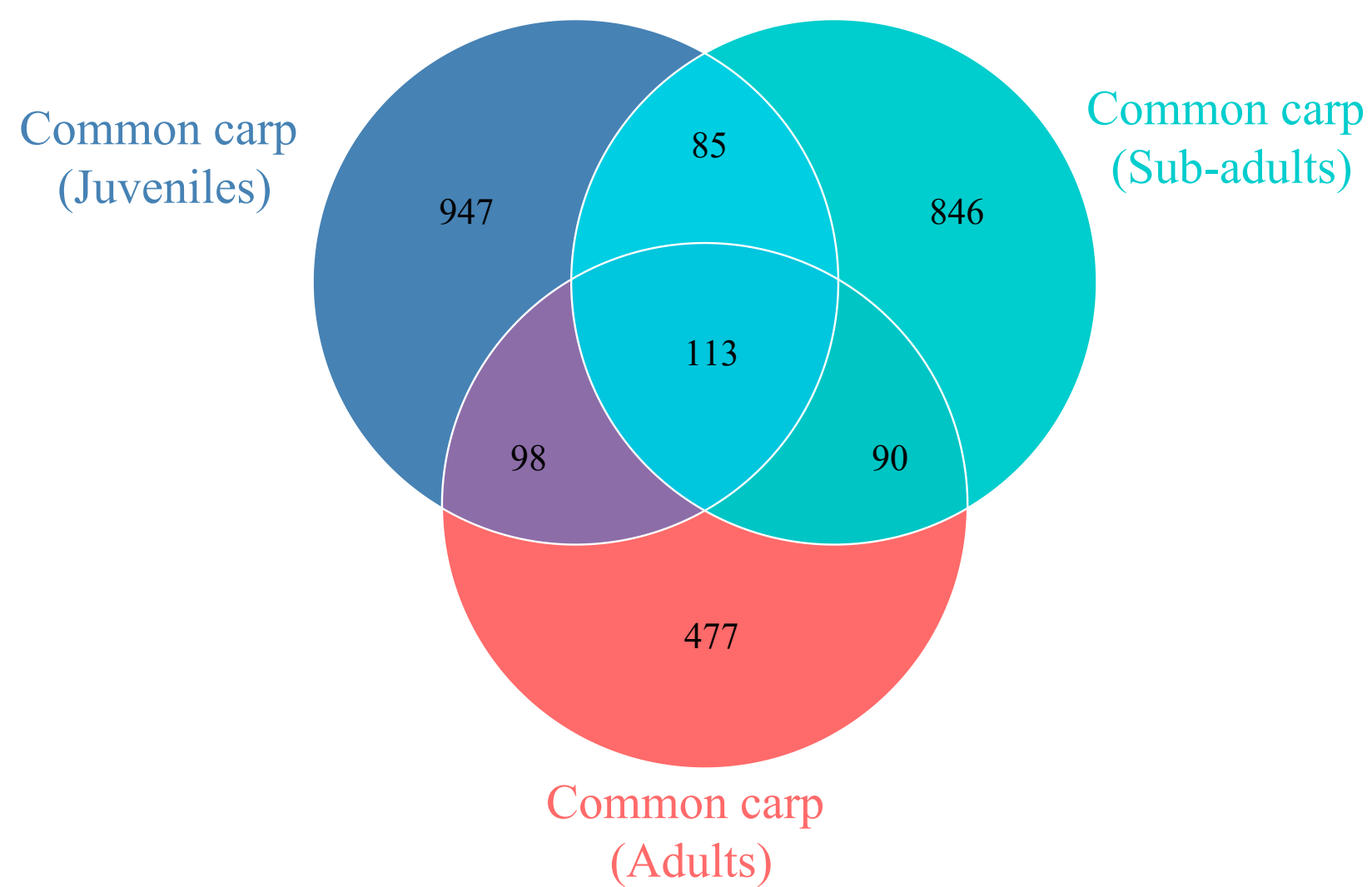

B

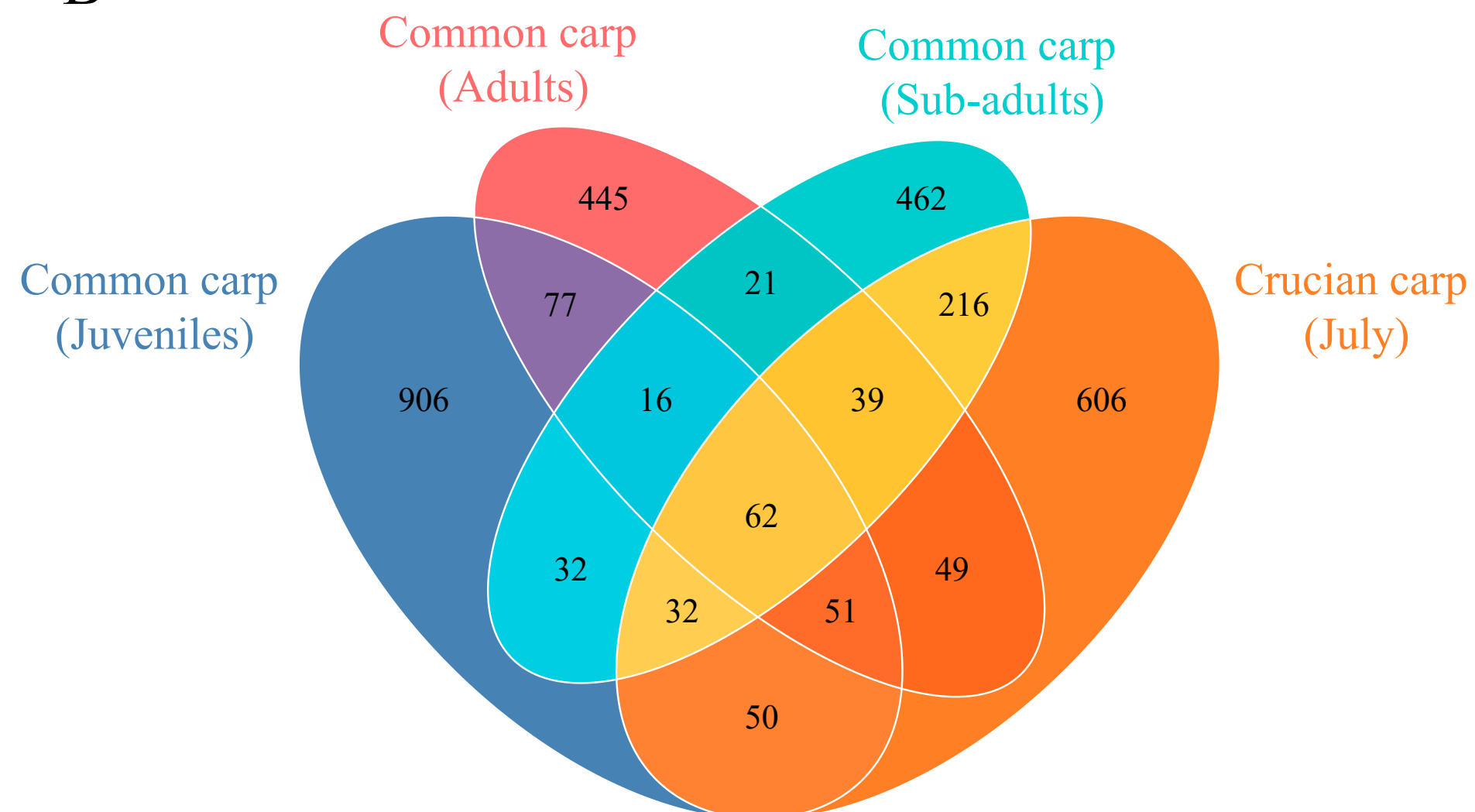

C

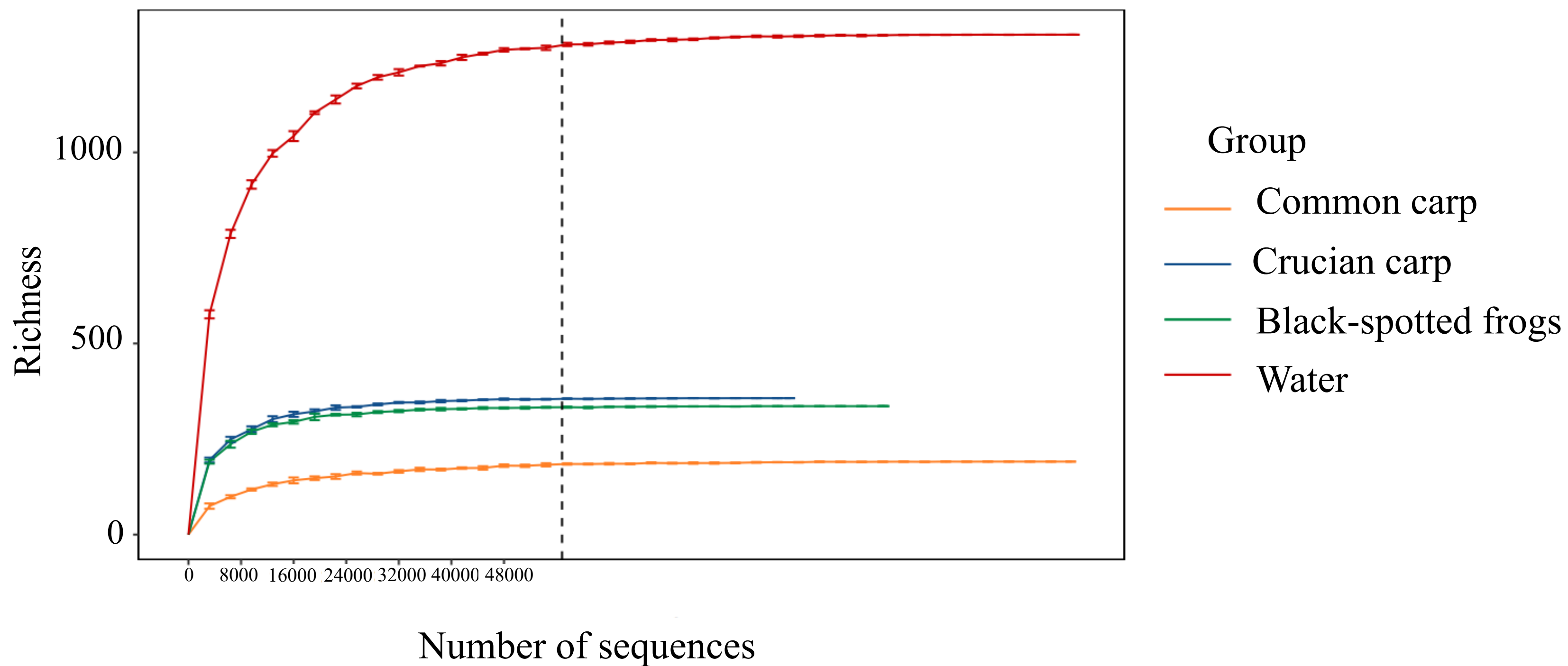

Supplement: Supplementary file 1 [file microorganisms-10-02373-s001.zip › Supplementary Figure S1.pdf]

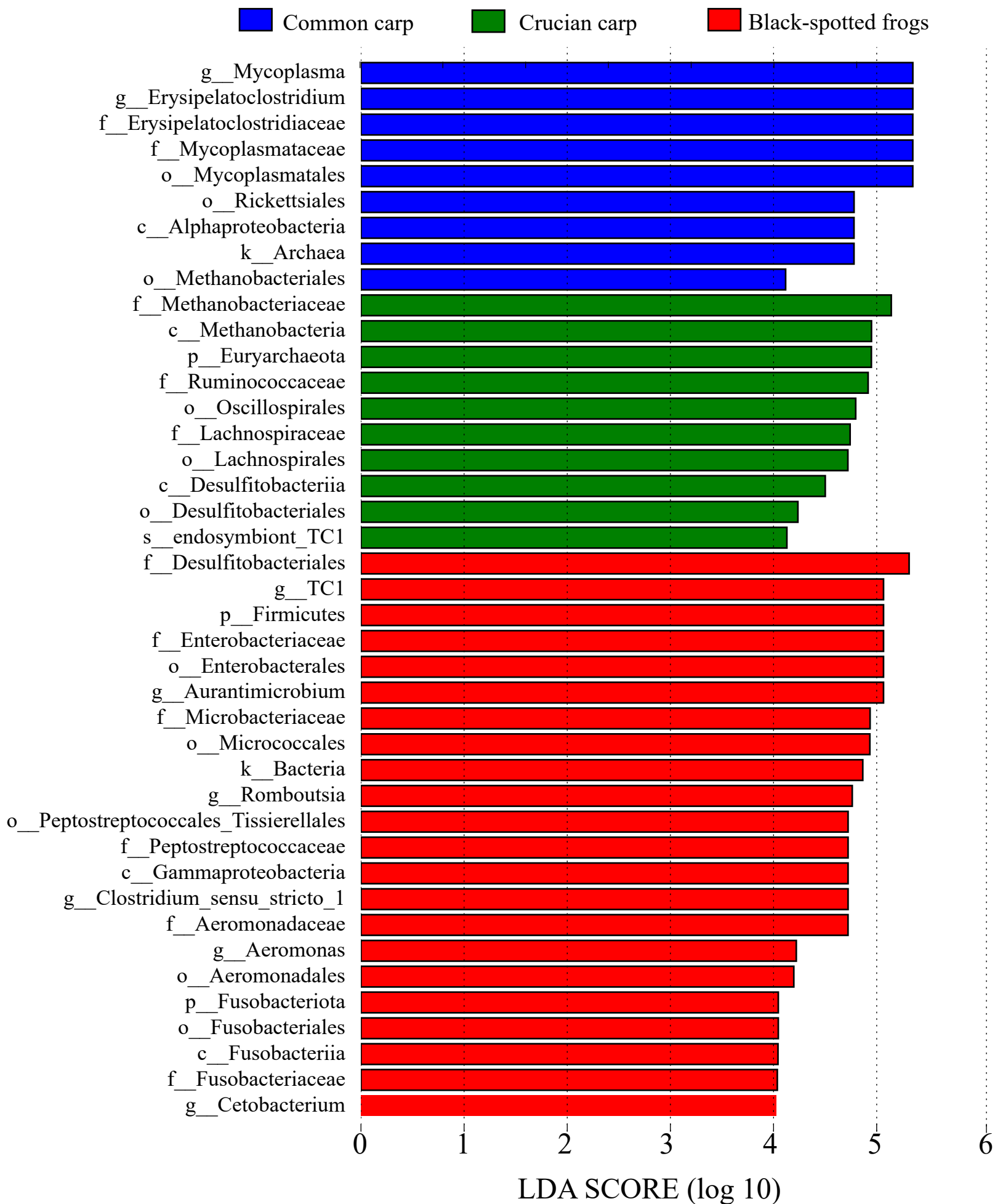

Supplement: Supplementary file 1 [file microorganisms-10-02373-s001.zip › Supplementary Figure S2.pdf]

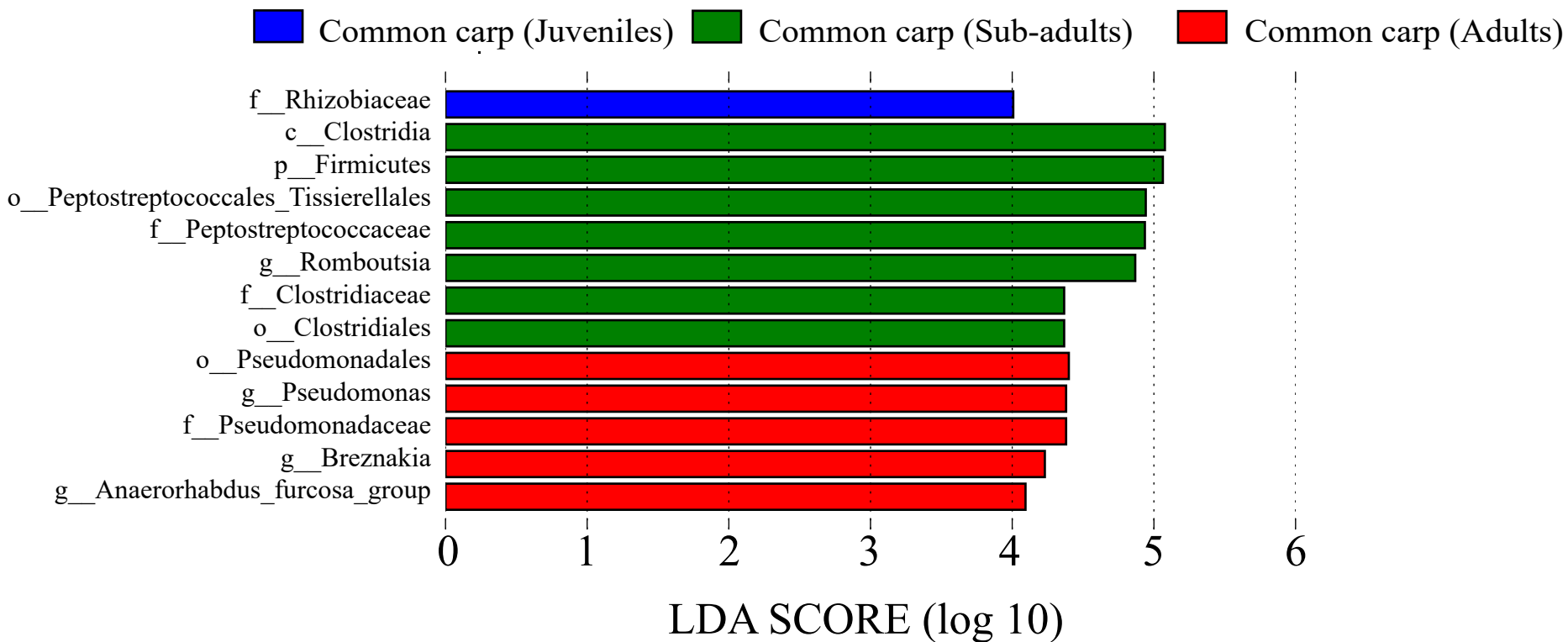

Supplement: Supplementary file 1 [file microorganisms-10-02373-s001.zip › Supplementary Figure S3.pdf]
